# “We're actually more of a likely ally than an unlikely ally”: relationships between syringe services programs and law enforcement

**DOI:** 10.1186/s12954-021-00515-2

**Published:** 2021-08-04

**Authors:** Carol Y. Franco, Angela E. Lee-Winn, Sara Brandspigel, Musheng L. Alishahi, Ashley Brooks-Russell

**Affiliations:** 1 Prevention Research Center for Family and Child Health, Department of Pediatrics, University of Colorado Anschutz Medical Campus, East 17th Avenue, Aurora, CO 1312180045 USA; 2 Program for Injury Prevention, Education and Research, University of Colorado Anschutz Medical Campus, Aurora, USA; 3 Department of Epidemiology, Colorado School of Public Health, University of Colorado Anschutz Medical Campus, Aurora, USA; 4 Department of Community and Behavioral Health, Colorado School of Public Health, University of Colorado Anschutz Medical Campus, Aurora, USA; 5 Department of Health Systems, Management and Policy, Colorado School of Public Health, University of Colorado Anschutz Medical Campus, Aurora, USA

**Keywords:** Syringe service programs, Needle exchange, Law enforcement, Harm reduction, Qualitative research

## Abstract

**Background:**

Syringe services programs provide sterile injection supplies and a range of health services (e.g., HIV and HEP-C testing, overdose prevention education, provision of naloxone) to a hard-to-reach population, including people who use drugs, aiming to prevent the transmission of infectious diseases.

**Methods:**

We performed a qualitative needs assessment of existing syringe services programs in the state of Colorado in 2018–2019 to describe—their activities, needs, and barriers. Using a phenomenological approach, we performed semi-structured interviews with key program staff of syringe services programs (*n* = 11). All interviews were digitally recorded, transcribed, and validated. A data-driven iterative approach was used by researchers to develop a coding scheme to organize the data into major themes found across interviews. Memos were written to synthesize main themes.

**Results:**

Nearly all the syringe program staff discussed their relationships with law enforcement at length. All syringe program staff viewed having a positive relationship with law enforcement as critical to the success of their program. Main factors that influence the quality of relationships between syringe services programs and law enforcement included: (1) alignment in agency culture, (2) support from law enforcement leadership, (3) police officers’ participation and compliance with the Law Enforcement Assisted Diversion (LEAD) program, which provides intensive case management for low-level drug offenders, and (4) implementation of the “Needle-Stick Prevention Law” and Drug Paraphernalia Law Exemption. All syringe program staff expressed a strong desire to have positive relationships with law enforcement and described how a collaborative working relationship was critical to the success of their programs.

**Conclusions:**

Our findings reveal effective strategies to foster relationships between syringe services programs and law enforcement as well as key barriers to address. The need exists for both syringe services programs and law enforcement to devote time and resources to build a strong, positive partnership. Having such positive relationships with law enforcement has positive implications for syringe services program clients, including law enforcement being less likely to ticket persons for having used syringes, and encourage people who use drugs to seek services from syringe services programs, which can then lead them to other resources, such as housing, wound care, and substance use treatment programs.

## Background

Syringe services programs (SSPs), also commonly referred to as syringe service sites, syringe exchange, syringe access, or needle exchange programs, aim to prevent the transmission of infectious diseases by providing sterile injection supplies and a range of other services to people who use drugs (PWUD) and have shown to be effective in reducing the spread of viral hepatitis and HIV [1–5]. SSPs take a harm-reduction public health approach, which aims to reduce the negative health effects of substance use [6]. In this context, SSPs focus on prevention of the primary sources of morbidity and mortality associated with injection drug use, including preventing infections, transmission of infectious diseases and overdose deaths, rather than abstinence from drug use. Despite the evidence for successful prevention of infectious diseases and other positive public health outcomes, the harm reduction approach is not universally adopted in public policies [2].

There is a growing prescence of SSPs in the US, currently operating in 39 states and D.C. [7], yet there remains variation in state laws restricting the purchase and possession of sterile syringes [8]. For instance, some states have decriminalized syringe possession or purchase, while others have allowed exemptions from drug paraphernalia charges [9]. Some SSPs started with controversial beginnings, in defiance of state laws banning the distribution of clean needles. For example, in Colorado, the setting of our study, the first syringe exchange program began in 1989, as a non-state sanctioned initiative to address the spread of HIV in one county [10]. Unlawful distribution of syringes continued until 2010, when the Colorado Governor signed a law legalizing these programs statewide (C.R. S. §25-1-520), [11]. Given these beginnings, it is not surprising that there could be discordance between SSP activities and law enforcement practices.

Prior studies have described how law enforcement actions can have a negative influence on the ability of a SSP to provide services [12–14]. This could be by direct interference with operations and access to SSPs or through other practices such as charging PWUD with paraphenalia in contradiction to the law [14–17]. Negative interactions with law enforcement can deter PWUD from using SSP services [18]. One reason for law enforcement practices that undermine harm reduction programs operated by SSPs could be lack of awareness on the part of law enforcement and perception that the harm reduction approaches are counterproductive [19–21].

However, the public health principles of harm reduction can be applied to law enforcement and policing [22, 23]. Identifying effective means of communication, including having tailored in-service training with police officers have proven effective in obtaining buy-in from law enforcement [20, 23-25]. There is evidence that law enforcement, when supportive of public health efforts, can facilitate participation by referring PWUD into the program [26]. There are harm reduction policing iniativies being adopted across the USA such as Law Enforcement Assisted Diversion (LEAD) programs in which police officers forgo the normal response of engaging a PWUD in the criminal justice system in favor of referring people into support systems and treatment programs [27]. In Seattle, Washington, this approach has been found to be successful in reducing recidivism and other positive outcomes [28].

Given the importance of the role of law enforcement professionals in the implementation of successful SSPs, we sought to better understand the nature of current relationships between SSP and law enforcement agencies in Colorado, and learn about factors that facilitate and hinder a collaborative relationship and adoption of a harm reduction approach by law enforcement. This study builds on the existing literature in the context of a US state with exemptions for drug paraphernalia charges.

## Methods

### Study design and population

In our qualitative study, we used a phenomenological approach to examine the relationships between SSPs and local law enforcement agencies and personnel. This approach allowed us to explore the phenomenon of interactions, engagement, and personal experiences between law enforcement and SSP staff. We conducted semi-structured interviews with key program staff at all legislated syringe service sites in the state of Colorado (*n* = 11 SSPs, *n* = 12 SSP staff, 2 from one site interviewed together). SSP staff included Executive Directors, program managers, and program staff members. As of 2019, there were 11 SSPs operating in eight counties across the state. The interviews were part of a broader needs assessment of currently operating programs. The goal of the needs assessment was to report on current activities of legislated SSPs currently operating in the state of Colorado to identify the needs for operating and barriers to effectively serving SSP clients.

We developed a semi-structured interview guide to understand how SSPs define, determine and/or measure their impact on the service population; SSPs’ knowledge of community perceptions of their program and services they provide; SSPs’ communication and/or collaboration with boards of health, county commissioners/city council, district attorneys, and law enforcement. We conducted interviews using broad lines of questioning and introduced additional questions during the interview process as relevant information was revealed by the interviewee. We asked participants (1) In what ways do you engage with law enforcement, and (2) Do you have a sense of how they view your program and the services you provide? These broad lines of questioning allowed us to further probe on SSPs staffs’ interactions and relationships with law enforcement, and how these relationships impact their ability to serve SSP clients. It also allowed SSP staff to share experiences between SSP clients and law enforcement during contact at a SSP agency.

### Recruitment and data collection

We received contact information for the primary contact for each SSP from the Colorado Department of Public Health and Environment (CDPHE). Primary contacts included program coordinators, directors, and health educators. Interviews were conducted either in-person (*n* = 9) or by videoconference (*n* = 2) depending on availability of the interviewee and their location. Participants were read a consent form by the interviewer and participants provided a verbal consent.

In-person interviews were recorded using a digital voice recorder (Olympus WS-852), and videoconference interviews were recorded using the Zoom application. Interviews lasted between 37 min to 1 h and 54 min. The interviews were led by members of the research team with experience in collective qualitative data (CF, SB, MA). Interview recordings were professionally transcribed via a third-party (TranscribeMe, San Francisco, CA), validated by members of the research team, and analyzed with qualitative analysis software NVivo11 (QSR International Pty Ltd, Doncaster, Victoria). Two independent study team members (CF, AL) conducted thematic analysis using data-driven, iterative process which allowed us to develop a coding scheme. Coding consistency checks were done between coders (Kappa coefficient > 0.80 [29]) and resolved discrepancies through discussion. After coding, memos were written to synthesize and categorize data into broader themes found among all participants. This process revealed two major themes, (1) The value of relationships between SSP and enforcement, and (2) Factors that influence the quality of relationships. We identified four subthemes of factors that influence relationships between SSP and law enforcement, including (1) Law enforcement culture, (2) Support from law enforcement leadership, (3) Law enforcement participation in the LEAD program, and (4) Law enforcements’ implementation of local laws related to SSPs and syringe users.

Data collection, analysis, and reporting followed guidelines established by Consolidated Criteria for Reporting Qualitative Research (COREQ) [30]. We present supporting quotes from SSPs staff indented and in italics.

The data collection procedures were reviewed by the Colorado Multiple Institutional Review Board and determined to be a quality improvement project and reviewed by the Institutional Review Board at the CDPHE.

## Results

### Overview of variation in relationships with law enforcement

Participants were asked about their interactions with law enforcement agencies, and all SSP staff members discussed at length the relationship with law enforcement. All SSP staff viewed having a positive relationship with law enforcement as important to the success of their program and having a negative or non-existent relationship with law enforcement as a barrier to the program and potentially having a negative impact on SSP clients. The degree of engagement and the type of collaboration with law enforcement varied by site. Relationships ranged from having a partnership with a high degree of interactions from both parties, having no relationship (evidenced by little coordination or communication), to having a hostile or confrontational relationship.

### Value of relationships

Programs that described having a positive relationship shared that the partnership had positive implications for SSPs and clients including facilitating referrals of clients into social services, working with SSPs to reduce syringe litter, and reducing re-offenses in the criminal justice system. Likewise, SSP staff stated that law enforcement agencies viewed SSPs as a resource for PWUD that provided critical services for this population in their community, and thus valued SSPs and supported their mission. Many of the SSP staff described the implications on SSP clients and the broader community when SSPs and law enforcement viewed each other positively and as a valuable resource, such as getting community members into social services. A SSP director detailed the positive and collaborative nature of their relationship with law enforcement, stating, “We have had very longstanding good relationships with law enforcement agencies… We work very closely with them on trying to do a comprehensive approach to substance use. And so, when we span the full spectrum, from prevention, early intervention, treatment, harm-reduction… we're trying to have a collective impact approach where we're reducing the negative impact of substance use in general.”

A few SSPs staff described collaborating with law enforcement in community efforts, such as holding syringe litter pick-up days in which SSPs and law enforcement partnered to clean up local areas with used syringes. Several SSPs staff also reported that local law enforcement who view SSPs positively will refer PWUD they encounter while performing their normal duties to SSP programs or other local resources instead of criminalizing them. SSP staff that reported a lack of collaboration with law enforcement expressed a desire to do so and acknowledged the potential benefits of having a positive relationship with agencies, including being able to learn from each other and working together to best serve substance users in their community. This lack of collaboration stemmed from law enforcement personnel’s lack of valuing SSP programs and a lack of understanding of the services provided, including services to the broader community such as HIV testing and referrals to services. A shared understanding and implementation of a harm reduction approach were described as factors influencing mutual respect and their perception of value to community members.

### Factors that influence quality of relationships

Several themes emerged as factors that influence the quality of relationships between SSPs and law enforcement. These include (1) alignment in agency cultures in adopting a harm reduction model, (2) support from law enforcement leadership, (3) police officers’ participation and compliance with the Law Enforcement Assisted Diversion (LEAD) program, and (4) variation in the implementation of laws, including the “Needle-Stick Prevention Law.” Ongoing communication between SSPs and law enforcement was a critical factor in all aspects of maintaining a positive relationship with law enforcement agencies.

#### Alignment in agency culture

Almost all SSP staff suggested that law enforcement agency culture, beliefs, or attitudes toward harm reduction approaches influenced law enforcement perception of SSP services, the relationship between SSPs and law enforcement, and had implications for SSP clients. Some SSP staff expressed that law enforcement’s positive endorsement of the harm reduction model and an agency culture marked by reduced biases toward people with substance use dependence supported a positive relationship between SSPs and law enforcement agencies. The partnership then led to law enforcement providing more support and resources for SSP clients, specifically PWUD. Alignment in attitudes and beliefs consisted of law enforcement officers viewing SSPs as a resource for PWUD to reduce the spread of disease, access treatment, and reduce the likelihood of needle-stick injuries for law enforcement. Those who did not share this belief viewed SSPs as a means to enable substance use and accelerate syringe litter in their community.

Through a shared philosophy of harm reduction, either through a collective agency culture or by individual police officers, SSPs and law enforcement were able to have a positive working relationship. There were several examples of how SSP and law enforcement could work together. For example, some SSPs conducted trainings for law enforcement and educated them on the benefits of using a harm reduction model to address substance use and the spread of disease. These trainings led to a change in perspective among law enforcement officers and assisted in positive collaboration and/or engagement. One SSP has representation of law enforcement on their board of directors and as a result has police officers regularly visit the site for tours and law enforcement refer potential PWUD clients to the SSP when on duty. Another SSP staff observed that law enforcement representatives attend opioid prevention coalition meetings. Many SSP staff noted that having an established positive relationship and the use of a harm reduction model with law enforcement leads to better communication, including identifying ways to better serve the community. A SSP staff member from one site recalled, “They [law enforcement] worked really closely with us in learning about the Law Enforcement-Assisted Diversion program, and they are now harm-reduction advocates themselves. And I am sometimes in a meeting and it's the [law enforcement] staff who are correcting people about harm reduction [laughter]… Bringing to people's attention like, "Oh, the only end goal is not treatment. We also need to think about harm reduction and keeping people safe when they're not actively using…”.

Law enforcement who did not agree with a harm reduction approach, labeled SSPs as “enablers,” according to several SSP program staff. They reported that this was a common and problematic perception that can have negative consequences for SSP clients and PWUD. A few SSP staff mentioned that law enforcement’s pre-existing negative perceptions toward PWUD and SSPs often lead law enforcement to ignore the state-wide law to protect SSP clients. They attributed such negative law enforcement culture to misunderstanding of SSPs’ role in the community. The belief that SSPs are “enablers” of continued drug use or directly providing illegal substances, such as fentanyl, were common misperceptions. One program manager stated, for example, “I think they view what we do as enabling and giving people the tools to do things illegally,” while another staff member from a different program advised, “I don't think that they view us as a spectrum of treatment or a spectrum of care. I think that a lot of it really boils down to the perception of the people who use drugs and that anybody who tries to help them is also bad.”

SSP staff suggested this is more common in law enforcement agencies having a “conservative” agency culture. One SSP staff member, for example, mentioned how one district attorney brought false and misleading evidence against the SSP to highlight how SSPs “enable” more drug use among their clients. These shared beliefs among law enforcement agencies have negative implications for SSP clients, as described by several SSP, whereby having a conservative agency culture was believed to have contributed to the charges placed on SSP clients having syringes on their person, reluctance of clients to seek help from police or other emergency personnel when their or someone else’s safety is endangered. One SSP manager described how clients are afraid to contact emergency services because police officers would arrest them instead of providing help, demonstrating an agency culture of criminalization of substance-using people instead of viewing them as people who need help or resources, stating “All of our people feel terrified… They don't feel comfortable to call emergency services because they know that the cops are going to come.”

#### Support from law enforcement leadership

Most sites discussing the role of law enforcement leadership discussed having positive relationships and that these relationships had implications for their program and the clients they are serving. Some SSP staff mentioned that positive endorsement and/or a positive relationship with law enforcement leadership was often a result of ongoing communications between SSP and law enforcement leaders, such as the Chief of Police, who would serve as a champion for SSP and their mission in reducing the spread of disease. SSPs were able to receive support from leadership by communicating the goals of SSPs with leadership, educating leaders of the role of SSPs on a spectrum of care, and their efforts to reduce syringe litter. Law enforcement leaders who advocated for SSPs were perceived as influencing the overall law enforcement agency culture and influencing the attitudes, beliefs, and behaviors of individual officers. Partnerships between law enforcement leadership and SSP staff also led to additional training for officers, including laws surrounding harm reduction and paraphernalia charges to SSP clients. A program manager from one SSP described communication with law enforcement leadership, and the need to educate officers on current laws surrounding syringes, “When we mentioned to them that we're seeing an uptick in people getting paraphernalia charges. They actually had in one of their chiefs meeting where all the chiefs of police come, they went and did a review of all the harm reduction laws that the officers need to be aware of and how they need to not be filing paraphernalia charges for participants [SSP client] of our program, and they are making sure that our local law enforcement are abiding to harm reduction laws.”

#### LEAD program participation

The Law Enforcement Assisted Diversion (LEAD) is a program that provides intensive case management for low-level drug offenders. Established in Seattle, WA, previous studies have shown how the LEAD results in a reduction in recidivism by diverting people to social services instead of incarceration [28]. The LEAD program offers an alternative route for low-level drug offenses that provides case management and connection with resources rather than jail and prosecution. The LEAD programs are operating in four counties in Colorado that also have SSPs [31], and nearly all these SSP staff noted this in their interviews that LEAD is a positive program in their community. Some SSP staff noted that LEAD is part of a changing culture in law enforcement around responding to drug use. One SSP staff member expressed the helpfulness of the program in giving law enforcement more options than arresting and putting individuals in jail when what they often need is services and treatment, stating “… But I think the officers here are more feeling like, ‘Oh, there's options we have besides just throwing someone in jail, they get back out, throwing them back in jail, they get back out.’ And so, I think that has helped giving law enforcement options. I mean it's still their decision whether they take someone to jail or not. But just to know that there are some options and support that they have.” Another SSP staff from a different site mentioned that the LEAD program is an opportunity to work in partnership with law enforcement, and that LEAD officers’ presence in the community provides a feeling of safety among PWUD and trust toward law enforcement. She said, “They've got folks that will come in here and volunteer fairly regularly. Again, that's an opportunity for them to make that face-to-face contact with the participants [SSP clients] within the program. They will wear their LEAD shirts and stuff in here. So that way, participants know that, ‘Hey, these guys are our reductionists. These guys aren't out to get you. These guys want to help you.’”.

Not all SSP staff however were successful at gaining buy-in from law enforcement to implement the LEAD program in their communities. A few SSP staff acknowledged challenges of successfully implementing and disseminating the LEAD program in their communities, including less systemic adoption of the LEAD program, strict criteria to implement the program, and unknown reasons for lack of buy-in from law enforcement leadership. A program manager from one site discussed the challenges in getting support for the implementation of LEAD in their community, saying, “They wanted to do LEAD, but the tops of law enforcement have set the exclusion criteria so strict that no one can really get into the program through them at that point.”

#### Varying implementation of laws

Many sites shared that officers do not adhere to laws that relate to ticketing of people for drug paraphernalia. According to state statute titled the Drug Paraphernalia Law Exemption (C.R.S. §18-18-425 through 18-18-430) [32], syringe exchange program clients are exempt from drug paraphernalia charges. A state Needle-Stick Prevention Law (C.R.S., §18-18-428) [32] also allows for an exception to drug paraphernalia charges if someone informs a law enforcement officer, prior to search, that they have a sharp object.

SSPs provide cards to clients to show they are exempt from drug paraphernalia charges. However, SSP clients are still sometimes ticketed, either due to an apparent lack of familiarity of the law, or in some cases, in apparent defiance of the law. Several SSP staff shared that law enforcement do not adhere to the needle stick law due to their personal beliefs toward PWUD or misunderstanding about the benefits of the law to officers. One SSP staff member implied the general lack of understanding of the Needle Stick law among some officers, suggesting, “So that law is meant to– it's meant to protect them.” A couple of sites noted the importance of framing the state statutes to protect law enforcement from being pricked by a used syringe while patting someone down. These staff shared that by presenting the law in a way that demonstrates its benefits to law enforcement officers, they would have more buy-in and adherence to the law by officers who have direct contact with SSP clients.

Among site staff who characterized their relationship with law enforcement as problematic, the key concern was around police officers ticketing their clients with paraphernalia charges. Despite having these regulations in place to protect SSP clients from being charged, program staff from several sites reported that clients had shown law enforcement their SSP Identification card (ID) and were still ticketed or charged for having paraphernalia. For example, one SSP staff member shared interactions involving clients producing a SSP card for officers, “We've had officers tear up our participants' [SSP client] syringe exchange cards, saying that harm reduction is not a real thing. This isn't legal… It's like once they see a syringe card, then they're like, ‘Okay, well, we want to search your car.’” A drug paraphernalia charge creates a cascade of events for SSP clients that can lead to negative outcomes, such as potential jail time. SSP staff often conduct outreach to law enforcement and district attorneys regarding inappropriate ticketing to dispute charges on behalf of the clients.

Several SSP staff described variation in law enforcement’s observance of the SSP ID cards, and a few claimed that law enforcement’s resistance to abiding by the Colorado statute were influenced by the officers’ personal views surrounding the statute. Similarly, a different site reported that their client also experienced resistance from law enforcement, and despite efforts to engage with law enforcement, they continue to give out tickets because of their negative views toward substance use and PWUD. Staff recounted an instance when officers were reported to dump out sharps containers to find syringes with residue in them, despite a SSP client having a SSP ID card on them. Another site discussed how law enforcement has become more accepting of the law and while many officers still ticket clients, it has become less frequent than when their site first opened.

## Discussion

The goal of our qualitative investigation was to identify challenges and barriers to operating SSPs and serving SSP clients. This study provides examples of challenges and successes in cooperating with law enforcement and the implications on SSP clients from the perspective of SSP staff. This qualitative study adds to existing literature on the relationship between law enforcement agencies and SSPs in the context of a US state with legislated SSPs exemptions for drug paraphernalia charges.

As part of this study, all study participants (SSP staff members) were asked about their relationships with law enforcement. Participants revealed a range of interactions and engagement with law enforcement, including positive collaborative relationship to adversarial relationships. Participants described the implications of the relationship for SSP clients, including unnecessary harassment and arrests by law enforcement. Participants describing a positive relationship with law enforcement discussed how programs and law enforcement shared similar views toward harm reduction initiatives, the need to reduce the spread of disease, and importance of connecting SSP clients to resources. Law enforcement training or education in harm reduction, communication between SSP staff and law enforcement agencies, and having a champion within law enforcement leadership were all factors contributing to a positive relationship. This is consistent with prior research that has shown the value of law enforcement training for building a collaborative relationship between law enforcement and SSPs [24]. Lack of a relationship was attributed to a lack of buy-in from law enforcement leadership and officers including a conservative political perspective and a lack of interest in establishing a relationship from leadership.

### Implications for policy and practice

Consistent with studies done in other communities in the USA, our findings suggest several activities or strategies are important to fostering relationships between police and SSPs, including SSPs to provide trainings, or encourage training, to law enforcement personnel on harm reduction models. Findings of prior research show that trainings on occupational safety information (e.g., needle-stick prevention) are acceptable to police and associated with a reduction in needle-stick injuries among law enforcement [12, 13, 33]. Future research could examine the trainings and training approaches that are most successful at supporting working relationships between SSP and with law enforcement. SSP can further demonstrate their commitment to supporting law enforcement by advocating for so-called needle stick laws. This is a discrete opportunity for SSPs to demonstrate they have the best interest of law enforcement officers in mind and can be advocates for law enforcement interests. A continuing focus on reducing syringe-related risks to law enforcement may be a pathway to demonstrate shared values.

Our findings suggest law enforcement professionals’ existing attitudes toward PWUD may supersede their adherence to the laws on SSP ID cards and result in inappropriate ticketing and charges. This calls for additional research on law enforcement professionals’ attitudes and beliefs toward SSPs and PWUD as well as their current training and understanding of the laws on SSP ID cards to expand our knowledge on building a successful working relationship between SSPs and law enforcement.

Finally, we found the adoption of the LEAD program is associated with a successful and supportive relationship between SSP and law enforcement [28]. The causal direction of this is unclear, it seems likely that it is bidirectional in that more supportive police districts choose to adopt the LEAD program which then reinforces a harm reduction approach. Thus, there remains the challenge of a SSP initiating or improving a relationship with the local enforcement leadership when a positive relationship does not yet exist.

## Limitations

Our findings are limited to existing SSPs in the State of Colorado; having data from one state with its unique community and legislative context may limit generalizability to states with differing policies. Detailed and nuanced results of our study, however, can inform practices and policies in other states with existing SSPs or are in the process of establishing a SSP in their community. Further research from the perspectives of law enforcement could also be beneficial as it may vary from our findings from the perspective of SSPs.

## Conclusion

All SSP staff expressed a strong desire to have positive relationships with law enforcement and described how a collaborative working relationship was critical to the success of their programs. Not all SSP staff, however, were fortunate in having a positive working relationship with law enforcement. Our findings suggest effective strategies to foster relationships between SSPs and law enforcement as well as key barriers to address. Our findings support the need for both SSPs and law enforcement to devote time and resources to build a strong, positive partnership. Having such positive relationships with law enforcement has implications for SSP clients, in that law enforcement will be less likely to ticket persons who get their syringes from SSPs, encourage PWUD to seek services from SSPs, which can then lead them to other resources, such as housing, wound care, and substance use treatment programs.

## Data Availability

De-identified data are available upon request to PI (Dr. Ashley Brooks-Russell) via email at ashley.brooks-russell@cuanschutz.edu.

## Abbreviations

SSP: Syringe services provider/program
HIV: Human immunodeficiency virus
CRS: Colorado revised statute
PWUD: People or Person/s who use drugs
LEAD: Law Enforcement Assisted Diversion
US: United States
CDPHE: Colorado Department of Public Health and Environment
CYF: Carol Y. Franco
SB: Sara Brandspigel
MA: Musheng L. Alishahi
CA: California
COREQ: Consolidated Criteria for Reporting Qualitative Research
ID: Identification
e.g.: Exempli gratia, for example

## References

[1] Aspinall EJ, Nambiar D, Goldberg DJ, Hickman M, Weir A, Van Velzen E, Hutchinson SJ (2014). Are needle and syringe programmes associated with a reduction in HIV transmission among people who inject drugs: a systematic review and meta-analysis. Int J Epidemiol.

[2] Centers for Disease Control & Prevention. HIV and injection drug use: Syringe services programs for HIV prevention. *Vital Signs* (2016).

[3] Fernandes RM, Cary M, Duarte G, Jesus G, Alarcão J, Torre C, Carneiro AV (2017). Effectiveness of needle and syringe Programmes in people who inject drugs–an overview of systematic reviews. BMC Public Health.

[4] Martin NK, Hickman M, Hutchinson SJ, Goldberg DJ, Vickerman P (2013). Combination interventions to prevent HCV transmission among people who inject drugs: modeling the impact of antiviral treatment, needle and syringe programs, and opiate substitution therapy. Clin Infect Dis.

[5] Platt L, Minozzi S, Reed J, Vickerman P, Hagan H, French C et al (2017) Needle syringe programmes and opioid substitution therapy for preventing hepatitis C transmission in people who inject drugs. Cochrane Database Syst Rev 9. 10.1002/14651858.CD012021.pub2 PMC5621373 28922449

[6] Stancliff S, Phillips BW, Maghsoudi N, Joseph H (2015). Harm reduction: front line public health. J Addict Dis.

[7] Kaiser Family Foundation. State Health Fscts: Sterile Syringe Exchange Programs (2018).

[8] LawAtlas. Syringe Service Program Laws. Retrieved from http://lawatlas.org/datasets/syringe-services-programs-laws (2019, August 1, 2019)

[9] LawAtlas. Syringe Distribution Laws. Retrieved from http://lawatlas.org/datasets/syringe-policies-laws-regulating-non-retail-distribution-of-drug-parapherna (2017, July 1, 2017)

[10] Asmar M. Why doesn't Colorado get the point of needle exchange programs? *Westword*. Retrieved from https://www.westword.com/news/why-doesnt-colorado-get-the-point-of-needle-exchange-programs-5103306 (2009)

[11] Colorado.gov. *Colorado Revised Statues 2018*. Retrieved from http://leg.colorado.gov/sites/default/files/images/olls/crs2018-title-25.pdf (2018)

[12] Beletsky L, Agrawal A, Moreau B, Kumar P, Weiss-Laxer N, Heimer R (2011). Police training to align law enforcement and HIV prevention: preliminary evidence from the field. Am J Public Health.

[13] Beletsky L, Grau LE, White E, Bowman S, Heimer R (2011). The roles of law, client race and program visibility in shaping police interference with the operation of US syringe exchange programs. Addiction.

[14] Small W, Kerr T, Charette J, Schechter MT, Spittal PM (2006). Impacts of intensified police activity on injection drug users: evidence from an ethnographic investigation. Int J Drug Policy.

[15] Bluthenthal RN, Kral AH, Erringer EA, Edlin BR (1999). Drug paraphernalia laws and injection-related infectious disease risk among drug injectors. J Drug Issues.

[16] Cohen J, Csete J (2006). As strong as the weakest pillar: harm reduction, law enforcement and human rights. Int J Drug Policy.

[17] Lazzarini Z, Bray S, Burns S (2002). Evaluating the impact of criminal laws on HIV risk behavior. J Law Med Ethics.

[18] Beletsky L, Cochrane J, Sawyer AL, Serio-Chapman C, Smelyanskaya M, Han J, Sherman SG (2015). Police encounters among needle exchange clients in Baltimore: drug law enforcement as a structural determinant of health. Am J Public Health.

[19] Beletsky L, Macalino GE, Burris S (2005). Attitudes of police officers towards syringe access, occupational needle-sticks, and drug use: a qualitative study of one city police department in the United States. Int J Drug Policy.

[20] Sightes E, Ray B, Paquet SR, Bailey K, Huynh P (2019). Police officer attitudes towards syringe service programming. Drug Alcohol Dependence.

[21] Wodak A, McLeod L (2008). The role of harm reduction in controlling HIV among injecting drug users. AIDS (London, England).

[22] Kammersgaard T (2019). Harm reduction policing: from drug law enforcement to protection. Contemp Drug Probl.

[23] Khorasheh T, Naraine R, Watson TM, Wright A, Kallio N, Strike C (2019). A scoping review of harm reduction training for police officers. Drug Alcohol Rev.

[24] Strike C, Watson TM (2017). Relationships between needle and syringe programs and police: an exploratory analysis of the potential role of in-service training. Drug Alcohol Depend.

[25] Watson TM, Bayoumi AM, Hopkins S, Wright A, Naraine R, Khorasheh T, Challacombe L, Strike C (2018). Creating and sustaining cooperative relationships between supervised injection services and police: a qualitative interview study of international stakeholders. Int J Drug Policy.

[26] DeBeck K, Wood E, Zhang R, Tyndall M, Montaner J, Kerr T (2008). Police and public health partnerships: evidence from the evaluation of Vancouver's supervised injection facility. Subst Abuse Treat Prevent Policy.

[27] Collins SE, Lonczak HS, Clifasefi SL (2017). Seattle’s Law Enforcement Assisted Diversion (LEAD): program effects on recidivism outcomes. Eval Program Plann.

[28] Clifasefi SL, Lonczak HS, Collins SE (2017). Seattle’s Law Enforcement Assisted Diversion (LEAD) program: within-subjects changes on housing, employment, and income/benefits outcomes and associations with recidivism. Crime Dlinq.

[29] McHugh ML (2012). Interrater reliability: the kappa statistic. Biochemia Medica Biochemia Medica.

[30] Tong A, Sainsbury P, Craig J (2007). Consolidated criteria for reporting qualitative research (COREQ): a 32-item checklist for interviews and focus groups. Int J Qual Health Care.

[31] Colorado Department of Human Services. Law Enforcement Assisted Diversion (LEAD) Program. Retrieved from https://www.colorado.gov/pacific/cdhs/law-enforcement-assisted-diversion-lead-program (2019, August, 2019)

[32] Colorado.gov. *Colorado public health harm reduction legislation*. Retrieved from https://www.colorado.gov/pacific/cdphe/colorado-public-health-harm-reduction-legislation (2019).

[33] Groseclose SL, Weinstein B, Jones TS, Valleroy LA, Fehrs LJ, Kassler WJ (1995). Impact of increased legal access to needles and syringes on practices of injecting-drug users and police officers–Connecticut, 1992–1993. J Acquir Immune Defic Syndr Human Retrovirol.

